# Gliomas Uncovered: A Deep Dive Into Immunohistochemical and Molecular Features From a Tertiary Care Facility Perspective

**DOI:** 10.7759/cureus.84522

**Published:** 2025-05-21

**Authors:** Pratiksha Mishra, Meenakshi Mohapatro, Kalyani P Gouda, Fakir C Munda, Lity Mohanty

**Affiliations:** 1 Pathology, Srirama Chandra Bhanj (SCB) Medical College and Hospital, Cuttack, IND; 2 Pathology and Laboratory Medicine, Postgraduate Institute of Medical Education and Research (PGIMER) Capital Hospital, Bhubaneswar, IND; 3 Pathology and Laboratory Medicine, Maharaja Krishna Chandra Gajapati (MKCG) Medical College and Hospital, Berhampur, IND

**Keywords:** cns tumors, gliomas, histomorphology, immunohistochemistry, molecular

## Abstract

Introduction

Central nervous system (CNS) tumors constitute a heterogeneous group of tumors comprising both benign and malignant populations. Central nervous system tumors carry substantial morbidity and mortality owing to their close anatomical relationship with vital neural structures. Although the exact etiology of CNS malignancies is not fully understood, several factors have been linked to an increased risk, such as genetic predisposition, previous exposure to ionizing radiation, and psychosocial stress. The tremendous increase in knowledge of the molecular markers for all CNS tumors during the last decade has allowed for a better understanding, evaluation, and treatment for the patients.

Materials and methods

This is a retrospective cross-sectional study carried out in the histopathology section of the Department of Pathology, Srirama Chandra Bhanj (SCB) Medical College and Hospital, Cuttack. The study lasted for 12 months, from June 2023 to June 2024. All the histopathologically confirmed cases of gliomas based on the recent 5th edition of the WHO classification were analyzed for further confirmation by molecular analyses. Clinical data (age, sex, grading), radiological investigations, and molecular studies were also done for the final diagnosis.

Results

A total of 66 histopathologically confirmed CNS lesions were analyzed, of which 34 (51.51%) were males and 32 (48.49%) were females. The majority of cases occurred in the 6th decade of life, with 13 cases observed. The maximum age recorded was 72 years, while the youngest patient was a one-month-old female. Twenty-one cases (31.8%) were classified as astrocytoma, IDH mutant, WHO grade 2, followed by WHO grade 4 (13 cases, 19.69%) and WHO grade 3 (nine cases, 13.6%).

Conclusion

This study highlights the importance of histopathological examination in conjunction with clinicoradiological features and molecular analysis to arrive at a final diagnosis especially for gliomas, glioneuronal and neuronal tumors. This combined ‘histo-molecular’ approach allows for a much more precise diagnosis of especially diffuse gliomas and all other CNS neoplasms.

## Introduction

Central nervous system (CNS) tumors constitute roughly 2% of all tumors, with incidence rates of five to 10 per 100,000 individuals in India, affecting both pediatric and adult populations [1-4]. Although the exact etiology of CNS malignancies is not fully understood, several factors have been linked to an increased risk, such as genetic predisposition, previous exposure to ionizing radiation, and psychosocial stress [2]. The pediatric population shows the second most common prevalence of CNS malignancies, only after hematopoietic neoplasms [5]. With the aim of understanding the different patterns of central nervous system neoplasms (CNS), including their relative frequencies, age and sex distributions, and clinicopathological presentation of the various tumors, this study was carried out in a tertiary care facility over a period of 12 months. This study highlights the importance of histopathological examination in conjunction with clinicoradiological features and molecular analysis in arriving at a final diagnosis. This combined ‘histo-molecular’ approach allows for a much more precise diagnosis of especially diffuse gliomas and all other CNS neoplasms.

## Materials and methods

Study design and setting

This study, designed as a retrospective cross-sectional analysis, was undertaken within the Department of Pathology at Srirama Chandra Bhanj (SCB) Medical College and Hospital, Cuttack. It lasted 12 months, commencing in June 2023 and concluding in June 2024. Ethical approval was obtained from the Institutional Ethics Committee at Srirama Chandra Bhanj Medical College and Hospital (approval number 1970/12-12-2024).

Sampling technique and data collection

The study employed purposive sampling. Detailed patient histories, along with clinico-radiological information and preoperative investigative findings where applicable, were meticulously gathered and analyzed across all cases. All the histopathologically confirmed cases of gliomas were further worked up for molecular analysis. Molecular analysis was performed to identify specific molecular features that define certain subsets of these neoplasms. The collected data were organized and analyzed to determine relative frequencies, as well as distributions across age and gender, which have been presented as numerical values or percentages.

Inclusion and exclusion criteria

This study includes 66 cases of gliomas that have been clinically and histopathologically confirmed, encompassing both benign and malignant forms. Non-neoplastic conditions and cases with insufficient biopsy material were excluded from the analysis.

Statistics

The study utilizes descriptive statistics to present data in terms of relative frequencies, distributions by age and gender, as well as numerical and percentage values.

## Results

Table 1 displays the gender distribution of gliomas identified in our center. Males constituted a larger portion, with 34 cases accounting for 51.51% of the total, while females comprised 32 cases, representing 48.49% of the total. This data highlights a higher prevalence of CNS neoplasms in males compared to females within the studied group, with a female-to-male ratio of 1.06:1.

**Table 1:** Gender distribution of cases

Gender	Number of Cases	%
Male	34	51.51%
Female	32	48.49%
Total	66	100

Table 2 categorizes all 66 cases of gliomas, glioneuronal, and neuronal tumors based on age distribution. Nine cases (13.63%) were identified in the <10 years group. The 11-20 years group included six cases (9%), 21-30 years group had seven cases (10.6%), 31-40 years group comprised 11 cases (16.66%), 41-50 years group contained 12 cases (18.18%), 51-60 years group encompassed 13 cases (19.69%), 61-70 years group had six cases (9%), and the 71-80 years group included two cases (3.03%). Therefore, the distribution of CNS neoplasms exhibited a higher prevalence among adults aged 51-60 years (13 cases, 19.69%).

**Table 2:** Age wise distribution of cases

Age Group (Years)	Number of Cases	%
0-10	9	13.63
11-20	6	9.0
21-30	7	10.6
31-40	11	16.66
41-50	12	18.18
51-60	13	19.69
61-70	6	9.0
71-80	2	3.03
TOTAL	66	100

**Figure 1:**
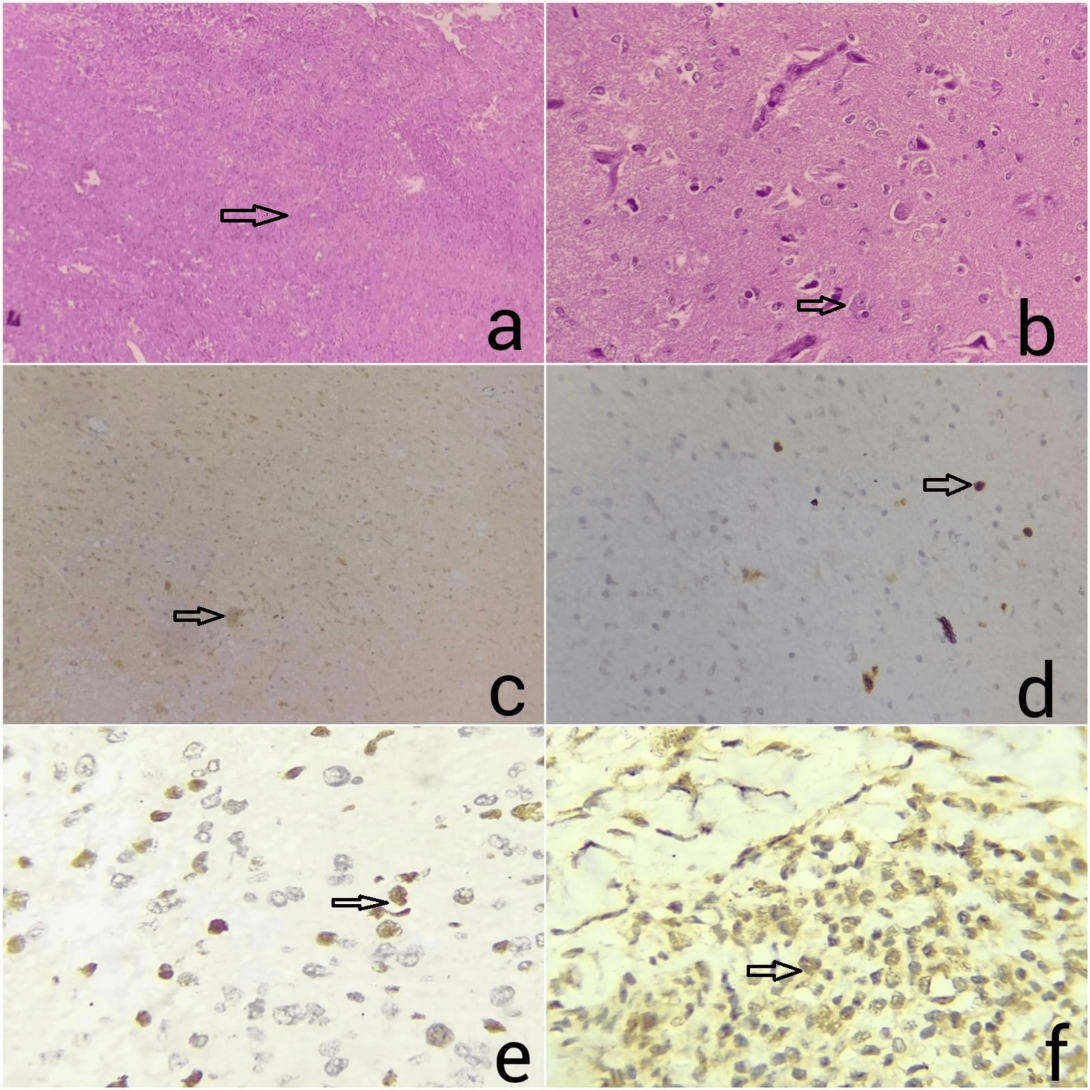
Immunohistochemistry (IHC) results *IDH* mutant Astrocytoma, WHO grade IV : (a) Microphotograph shows diffusely infiltrating tumor cells, (b) Diffusely infiltrating tumor cells characterized by round to oval cells, scanty amount of eosinophilic cytoplasm, irregular nuclear outlines, and prominent nucleoli, (c) IDH is positive in tumor cells (IHC, 400), (d) *Ki67* index is 15% (e) *ATRX* positive in tumor cells (IHC, 400X), (f) *P53* positive in tumor cells (IHC, 400X).

Table 3 categorizes all 66 cases of astrocytic neoplasms based on histologic subtypes. Twenty-one cases (31.8%) belonged to astrocytoma, *IDH* mutant, WHO grade 2, followed by WHO grade 4 (13 cases, 19.69%) and WHO grade 3 (nine cases, 13.6%). The most common pediatric CNS neoplasm was pilocytic astrocytoma (seven cases, 10.6%). Oligodendroglioma, *IDH* mutant, WHO grade 2, and grade 3 demonstrated four and three cases, respectively. The histomorphology of a 53-year-old female, as shown in Figures 1a-1f, was diagnosed as astrocytoma, WHO grade 4 *IDH* mutant, *ATRX* and *TP53* mutant case.

**Table 3:** Histological subtypes of astrocytic tumors based on IHC and molecular studies IHC: Immunohistochemistry.

S.No.	Histological subtypes	Grade	Total cases	Percentages
	Astrocytoma, *IDH* Mutant	WHO grade 2	21	31.8%
		WHO grade 3	9	13.6%
		WHO grade 4	13	19.69%
	Oligodendroglioma, *IDH* Mutant	WHO grade 2	4	6.06%
		WHO grade 3	3	4.54%
	Glioblastoma, *IDH* wildtype		7	10.6%
	Pilocytic astrocytoma		7	10.6%
	Pleomorphic xanthoastrocytoma		2	3.03%
Total cases: 66 (100%)				

**Figure 2:**
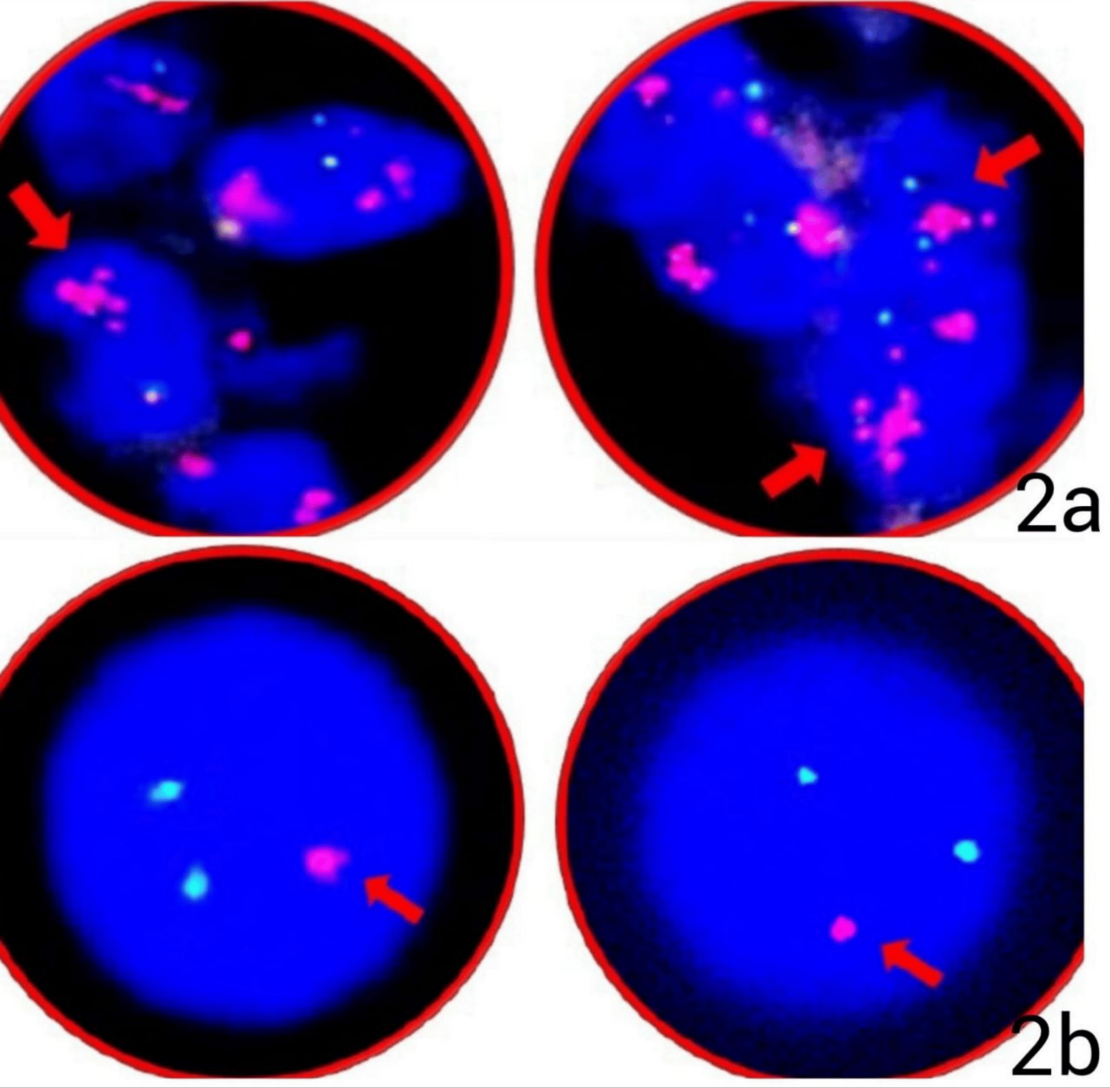
Histomorphology study image (a) Case 1 shows formalin-fixed, paraffin-embedded (FFPE) positive for *EGFR* gene mutation in a 60-year-old female, (b) Case 2, a 60-year-old male, presented positive for 1p36/ 19q13 co-deletion hypermethylation.

We extensively carried out a molecular study in all the astrocytic neoplasm cases, depicting the results of three cases in Table 4. Case 1, 60 years female with a provisional diagnosis of astrocytoma based on histomorphology and IHC, showed analyzed tumor cells in the sections presented normal copies for both *CEP7*/*EGFR* gene, however the ratio of *EGFR*/*CEP7* was >2.0 with an average *EGFR* gene copy number of >4.0 signals/cells, hence making it positive for *EGFR* gene mutation (Figure 2a). The analyzed formalin-fixed, paraffin-embedded (FFPE) DNA sample was also positive for MGMT gene promoter hypermethylation. Case 2, a 60-year-old male, showed the tumor section presented with positive for 1p36/ 19q13 co-deletion, and the analyzed FFPE DNA sample was also positive for *IDH1* gene, p.R132H (c.395G>A) variant (Figure 2b). Case 3 of a 36-year-old female showed the analyzed FFPE DNA sample to be positive for c.228C>T variant of the *TERT* gene, positive for the *IDH2* gene, p.R172M variant, and positive for MGMT gene promoter hypermethylation.

**Table 4:** The molecular features of three cases

S. No.	Age & Gender	Provisional diagnosis	*IDH1*, *IDH2*	1p36/19q13	CDKN2A/2B (9p21)	ATRX	TERT	*EGFR* Gene (7p11.2)	P53	MGMT
	60 Y F	Astrocytoma NOS	-	-	-	-	-	Amplified (Positive)	-	Positive
	60 Y M	Oligodendro-glioma	IDH1 positive	Positive	-	-	-	-	-	-
	36 Y F	Astrocytoma NOS	IDH2 positive	-	-	-	Positive	-	-	Positive

There was a single case of high-grade spinal ganglioglioma diagnosed in a 15-year-old male who showed dysplastic ganglion cells and numerous oligodendrocytic cells in a gliofibrillary background. Further molecular analysis showed the analyzed FFPE DNA sample to be positive for the *BRAF* (v600E) complex gene variant. The detected gene variant was c.1799_1800 TG>AA, thus confirming the same.

## Discussion

In developed countries, advances in molecular approaches have facilitated the development of targeted therapies, leading to improved patient outcomes [5]. Over the past year, we conducted a study to analyze the histomorphology, immunohistochemistry, and molecular characteristics of gliomas at our center.

Gliomas are the most common primary malignant neoplasms of the CNS, as also seen in our case. WHO Histological grading is done on the basis of mitosis, nuclear pleomorphism, necrosis, microvascular proliferation, or CDKN2A/2B deletion. *IDH1* or *IDH2* mutations have been identified in more than 70% of CNS neoplasms diagnosed as grade II and III astrocytoma, oligodendroglioma, oligoastrocytoma, and secondary glioblastoma. These mutations are rarely found in other brain tumors (e.g., primary glioblastoma and pilocytic astrocytoma), hence imparting a favourable prognosis. *IDH1* and *IDH2* mutations have been found in other non-CNS neoplasms, including cholangiocarcinoma, acute myeloid leukemia, chondrosarcoma, and angioimmunoblastic T-cell lymphoma, but the pathogenesis in these entities remains unclear [6-8]. Point mutations in the TERT promoter region create new transcription factor binding sites, thereby increasing the expression of the telomerase enzyme encoded by TERT. The most common mutations in the TERT promoter are C228T and C250T, which correspond to C>T transitions at genomic positions hg19/GCRh37 chr5:1295228 and chr5:1295250, respectively. In oligodendrogliomas, TERT mutations are linked to a poor prognosis in the absence of IDH mutations. Conversely, the absence of TERT promoter mutations combined with the presence of *IDH* mutations is indicative of astrocytoma [9-12]. The *ATRX* gene encodes the ATRX protein, a transcriptional regulator with two functional domains: the zinc finger domain, which acts as a transcription factor, and the helicase domain, which facilitates the unwinding of double-stranded DNA during transcription. Mutations in the ATRX protein result in downregulation of the α-globin locus, leading to thalassemia, and potentially contribute to gene expression abnormalities through disrupted transcription and chromatin structure, causing developmental malformations and intellectual disabilities [9-12].

Epigenetic silencing of the MGMT (O6-methylguanine DNA methyltransferase) gene via promoter methylation impairs DNA repair mechanisms and is associated with improved treatment responses and extended overall survival in glioma patients treated with alkylating agents. MGMT reverses guanine alkylation by transferring the alkyl group to its active site. Reduced MGMT expression induces DNA damage signaling and cell death. MGMT promoter methylation in glioblastoma enhances responsiveness to alkylating agents such as BCNU (carmustine) and temozolomide, contributing to progression-free survival [13-15]. *TP53*, a tumor suppressor gene, is the most commonly mutated gene in human malignancies. Evidence suggests it also has a major role in “gain of function mutation” [16,17].

*BRAF* mutation can occur in different ways, either inherited or later in life. BRAFv600E mutations have been seen in hairy cell leukemia, non-Hodgkin lymphoma, colorectal malignancies, malignant melanoma, papillary thyroid carcinoma, etc. [18,19]. Amongst CNS tumors, mutation is seen in pleomorphic xanthoastrocytomas, ganglioglioma (as seen in our case), dysembryoplastic neuroepithelial tumor, pilocytic astrocytomas, and very rarely in glioblastomas [19-20].

Hence, identifying molecular subtypes, particularly through large-scale sequencing efforts, has led to better classification of these tumors beyond traditional histopathological criteria. Specific mutations in genes such as *IDH1*, *TP53*, *ATRX*, and *EGFR*, among others, have been identified as drivers of tumorigenesis in gliomas. These mutations often alter critical cellular pathways (e.g., cell cycle regulation, DNA repair, and metabolism), targeting new molecular therapies.

## Conclusions

We emphasize the critical importance of integrating molecular studies with histopathological and immunohistochemical examinations to achieve accurate and definitive diagnoses. A structured pathology report, which includes detailed histopathological findings, immunohistochemical results, and molecular analyses, is essential. Understanding the molecular characteristics of CNS tumors is crucial for improving diagnosis, prognostication, and treatment strategies.
